# Rapid Screening of Glucocorticoid Receptor (GR) Effectors Using Cortisol-Detecting Sensor Cells

**DOI:** 10.3390/ijms22094747

**Published:** 2021-04-29

**Authors:** Jeahee Ryu, Euiyeon Lee, Chungwon Kang, Minhyeong Lee, Soyoun Kim, Seungil Park, Daeyeon Lee, Youngeun Kwon

**Affiliations:** Department of Biomedical Engineering, Dongguk University, Seoul 04620, Korea; annlove7@dongguk.edu (J.R.); euiyeon.lee@dongguk.edu (E.L.); iu8974@dgu.ac.kr (C.K.); 2017126656@dgu.ac.kr (M.L.); youn3256@gmail.com (S.K.); qnpd1234@gmail.com (S.P.); lwiam1216@gmail.com (D.L.)

**Keywords:** cortisol, glucocorticoid receptor (GR), GR effector, selective GR agonist, cell-based sensor, signal peptide reconstitution, conditional protein splicing

## Abstract

Cortisol, a stress hormone, plays key roles in mediating stress and anti-inflammatory responses. As abnormal cortisol levels can induce various adverse effects, screening cortisol and cortisol analogues is important for monitoring stress levels and for identifying drug candidates. A novel cell-based sensing system was adopted for rapid screening of cortisol and its functional analogues under complex cellular regulation. We used glucocorticoid receptor (GR) fused to a split intein which reconstituted with the counterpart to trigger conditional protein splicing (CPS) in the presence of targets. CPS generates functional signal peptides which promptly translocate the fluorescent cargo. The sensor cells exhibited exceptional performance in discriminating between the functional and structural analogues of cortisol with improved sensitivity. Essential oil extracts with stress relief activity were screened using the sensor cells to identify GR effectors. The sensor cells responded to peppermint oil, and L-limonene and L-menthol were identified as potential GR effectors from the major components of peppermint oil. Further analysis indicated L-limonene as a selective GR agonist (SEGRA) which is a potential anti-inflammatory agent as it attenuates proinflammatory responses without causing notable adverse effects of GR agonists.

## 1. Introduction

Physical or emotional stress results in a surge of chemicals in the human body, including the stress hormone cortisol. Cortisol is a glucocorticoid (GC), an adrenal steroid hormone, which helps the body to respond to stress appropriately. Cortisol plays a key role in maintaining homeostasis and metabolism in the human body and coordinating the immune system’s negative feedback mechanism to lower immune activity [1,2,3,4,5,6]. Abnormal cortisol levels can trigger various adverse symptoms including bone and muscle breakdown, fatigue, depression, pain, and memory impairments and lead to endocrine disorders such as Addison’s and Cushing’s diseases [7,8,9]. Therefore, the discovery of cortisol analogues has attracted considerable interest in drug development research [10,11]. For example, a cortisol-receptor agonist, dexamethasone (Dex), has been marketed as an anti-inflammatory agent for over 50 years; the cortisol-receptor antagonist, mifepristone (RU-486), is prescribed in the USA against hyperglycemia secondary to hypercortisolism in patients with Cushing disease.

**Scheme 1:**
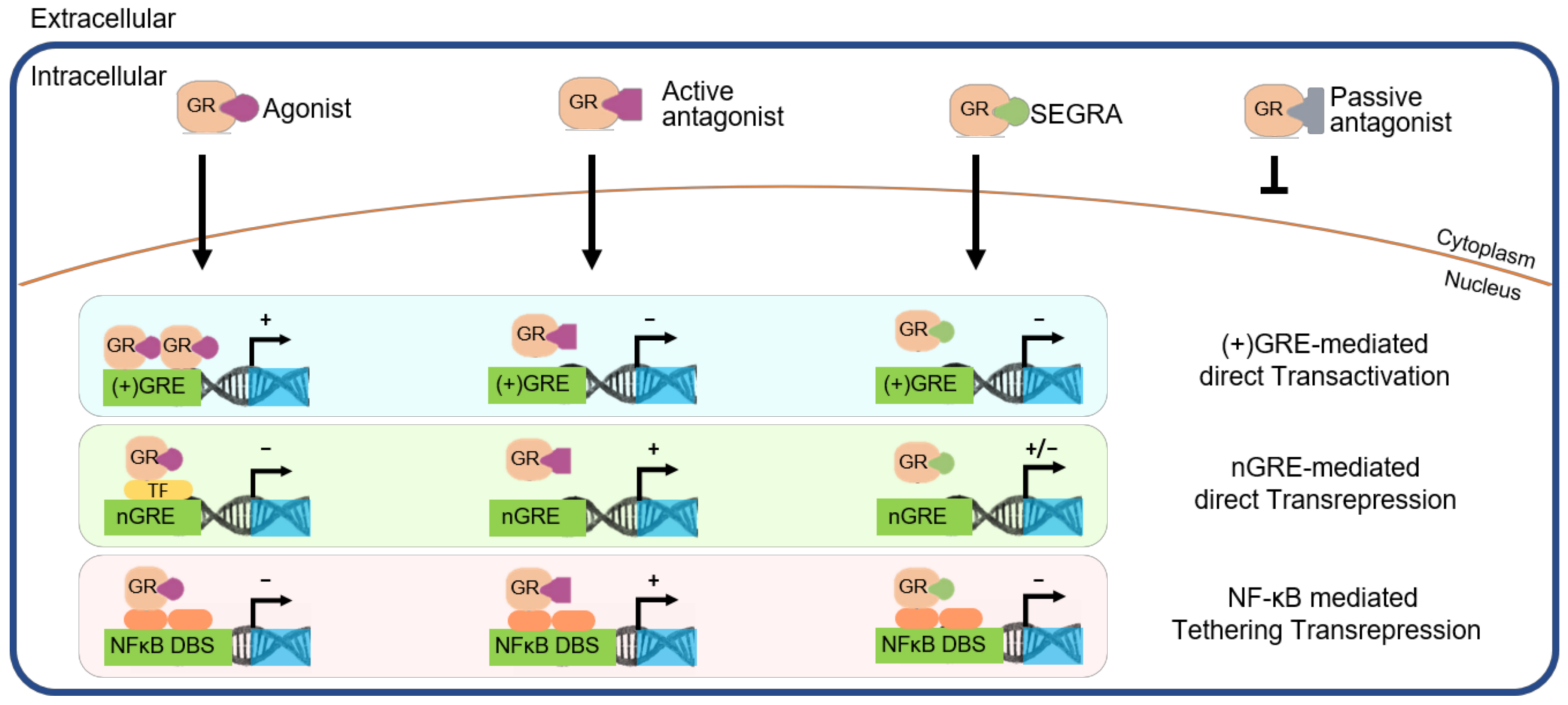
A schematic illustration of the molecular mechanisms by which glucocorticoid receptor (GR) regulates expression of its target genes. Plus (+) indicates stimulation of gene activation or repression, whereas minus (−) indicates no effect on stimulation or repression.

The effects of cortisol and cortisol analogues are mediated by glucocorticoid receptor (GR), which acts as a ligand-activated transcription factor. There are four major types of glucocorticoid receptor (GR) effectors, i.e., agonists, active antagonists, passive antagonists, and selective GR agonists (SEGRAs) (Scheme 1). GR agonists induce GR-mediated transactivation and transrepression, whereas active antagonists inhibit both GR-mediated transactivation and transrepression. SEGRAs function as activators of GR-mediated transrepression and inhibitors of GR-mediated transactivation. Passive antagonists bind to GR but inhibit its nuclear translocation. GR agonists are often considered to be potential drug candidates with strong anti-inflammatory activity by inducing strong anti-inflammatory gene such as MKP1, GILZ, IκB via their transactivation effects, but, severe adverse effects such as skin atrophy and muscle weakness are also reported when these agonists are used in high dose for prolonged period of time. Therefore, there is an urgent need to develop more selective GR agonists that maintain the anti-inflammatory activity, however, cause less adverse effects.

Several approaches have been used to screen for cortisol and its analogues, such as immunoassays and GR-competitor assays [12,13,14,15,16,17,18,19,20,21,22,23,24]. While immunoassays enable sensitive and rapid screening of cortisol, it is not capable of screening functional analogues as the sensing is affected via specific antigen-antibody recognition [12,13,18,20,21]. GR competitor assays allow screening of GR effectors, however, a few important aspects must be considered. First, the interactions between purified targets and probes may not be the same as those in biological fluids. Second, this assay cannot provide any information on GR’s response upon ligand binding [22]. Therefore, a sensing system that can perform function-based screening of cortisol analogues in biologically relevant environments is critical. Cell-based sensors offer an alternative sensing platform that can address the aforementioned shortcomings. Cell-based sensors are often fabricated as genetically encoded biosensors by using native receptors or enzymes as molecular recognition components and fluorescent or luminescent proteins as optical reporters [25,26,27]. In its simplest format, a fluorescence protein-tagged GR has previously been used to screen the movement of GR [28]. However, this approach is not suitable for screening of GR effectors as this type of sensor cells does not show adequate sensitivity to report subtle changes quantitatively, due to their high background.

**Scheme 2:**
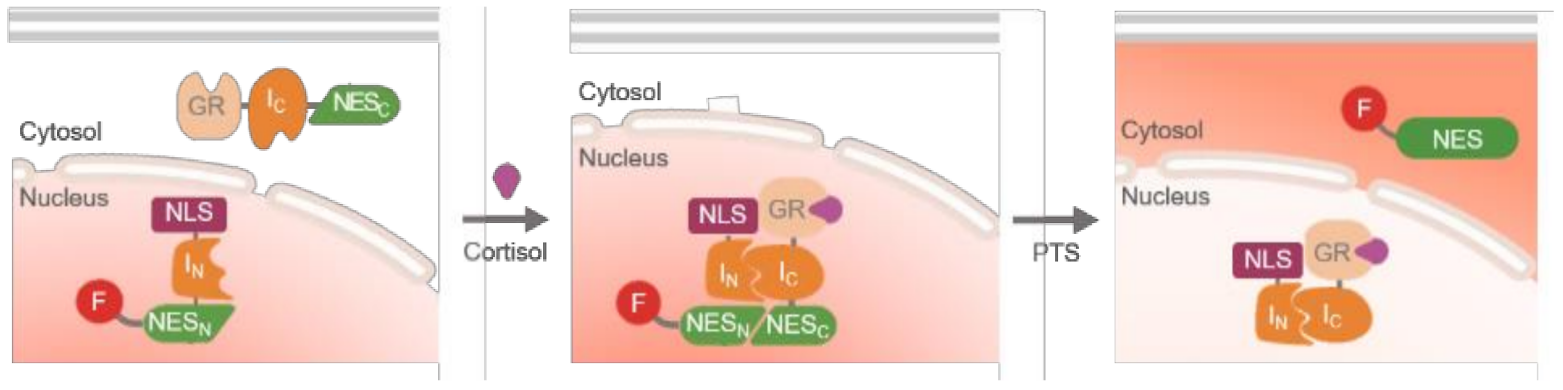
A schematic description of fluorescence translocation sensor cells. Two fusion proteins containing each split intein are initially located in two different compartments of a sensor cell to hinder the spontaneous protein trans-splicing. The target induces nuclear translocation of the glucocorticoid receptor (GR) containing fusion protein to initiate conditional protein splicing (CPS). The reconstituted signal peptide is instantly activated to deliver the fluorescent cargo into the cytosol to report the presence of the target.

In this study, we adopted cell-based sensors for the specific and sensitive screening of GR effectors. The intein-mediated conditional protein splicing (CPS) reaction was exploited to fabricate the sensor cells. GR effectors triggered CPS to generate reconstituted signal peptides. The signal peptides translocated the fluorescent cargo from the nucleus to cytosol to report the presence of target molecules (Scheme 2). For the screening of natural products, we engineered sensor cells, equipped with accelerated reporting capabilities that instantaneously activated after covalent bond formation, as the long response time severely limited the practical use of the sensor cells [29]. We evaluated the performance of the developed sensors and used them for rapid screening of cortisol analogues in various essential oils often used for stress relief. We further analyzed the newly identified active components in essential oils to elucidate their biological functions.

## 2. Results and Discussion

### 2.1. Design and Construction of a Genetically Encoded Biosensor for Cortisol Detection

**Figure 1:**
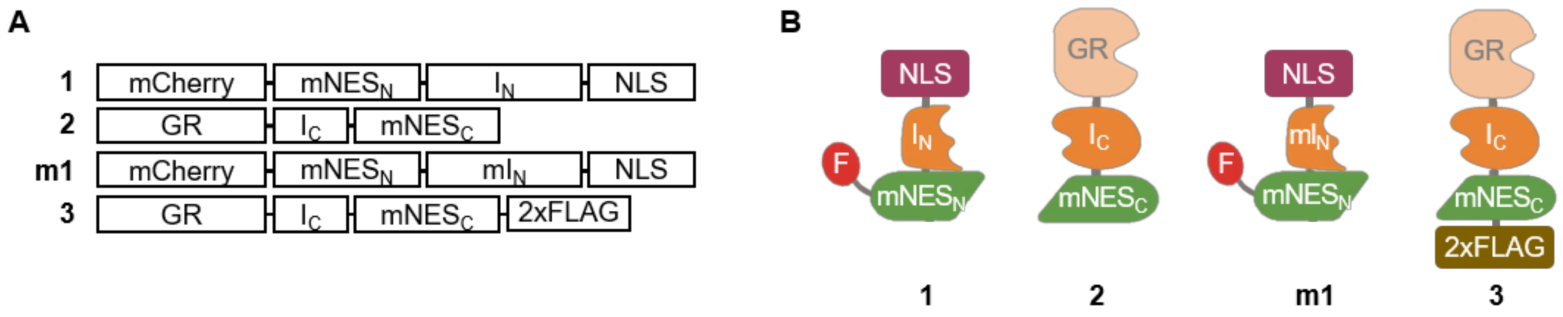
Fusion proteins used in this study. (**A**) Domain architecture of the DNA constructs of the fusion proteins containing N- or C-intein fragments; (**B**) a schematic illustration of fusion proteins **1**, **2**, **m1**, and **3**.

To screen the GR effectors, we designed a sensing system based on the well-studied pair of *Nostoc punctiforme (Npu)* DnaE split inteins, which mediate fast and efficient protein trans-splicing (PTS) reactions [30,31,32]. The key to our approach is that cortisol-bound GR translocates to the nucleus, triggering CPS to reconstitute the split signal peptide to transport the fluorescent cargo (Scheme 2). Two genetic constructs encoding fusion proteins **1** and **2** (mCherry-mNES_N_-I_N_-NLS and GR-I_C_-mNES_C_) were designed to generate the sensor cells (Figure 1). The modified NES (mNES) sequence (KVYPIILRL*CFN*LSL), derived from the human SARS corona virus ORF-9b protein, was split at position 9, and each fragment was used as the N- and C-extein in fusion proteins **1** and **2**, respectively (Figure S1) [33]. The red fluorescent mCherry protein was attached to the *N*-terminus of *N*-extein in protein **1** as an optical reporter. To obstruct the spontaneous PTS of *Npu* DnaE intein, the GR-containing protein **2** was localized in the cytoplasm, whereas the mCherry-containing protein **1** was positioned in the nucleus by using the C-terminal nuclear localization signal (NLS) peptide. The GR-containing protein **2** translocates to the nucleus upon cortisol binding to activate the PTS-generating active mNES that delivers the fluorescent cargo to cytoplasm. Variants of each fusion protein were also prepared. The splicing inactive mutant of **1** (protein **m1**) was prepared by introducing C1A mutation in I_N_ and used to monitor the necessity of PTS-mediated covalent bond formation in the activation of the split signal peptide. The 2xFLAG-tag introduced variant of **2** (protein **3**) was prepared for a Western blot analysis. The domain architectures of the gene constructs and fusion proteins are shown in Figure 1.

### 2.2. Detection of Cortisol Using the Cell-Based Sensor

**Figure 2:**
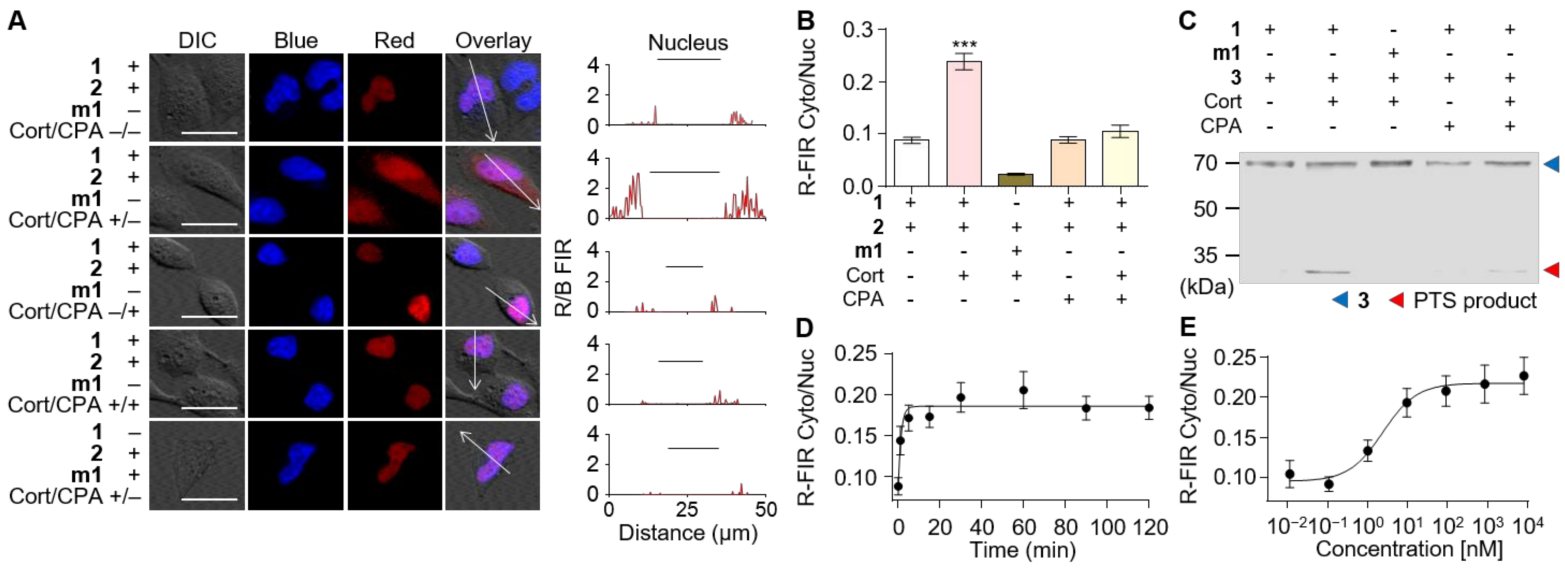
Detection of cortisol (Cort) stimulation using sensor cells based on the conditional protein splicing reaction and performance evaluation of sensor cells. (**A**) The sensor cells co-expressing proteins **1** and **2** show red fluorescence in the nucleus before stimulation (row 1), and the fluorescence signal translocates to the cytosol when sensor cells are challenged with Cort (row 2), the GR antagonist, cyproterone acetate (CPA), did not induce signal translocation (row 3) and instead inhibited Cort-mediated signal translocation (row 4), mock sensor cells containing fusion proteins **m1** and **2** did not show fluorescence translocation when challenged with Cort (row 5, scale bar = 25 μm); (**B**) the red fluorescence intensity ratios from the cytoplasm to nucleus (R-FIR Cyto/Nuc) are calculated, data were analyzed using one-way ANOVA using the Tukey multiple comparison test (*** *p* < 0.001); (**C**) the Western blot analysis showed the formation of CPS product in the presence of target from the sensor cells, suggesting covalent conjugation of the split-signal peptide; (**D**) the time-response curve demonstrates the sensor responses as early as 1 min after Cort stimulation, and R-FIR Cyto/Nuc plateaued approximately at 10 min; (**E**) dose-response fluorescence intensity curve showing the increase in the fluorescence intensity ratio with increasing Cort concentration, the limit of detection was 3.5 nM.

To create cortisol-detecting sensor cells, the genes encoding for fusion proteins **1** and **2** were inserted into a pBI-CMV1 vector containing two constitutive promoters, *P*_CMV IE_ and *P*_minCMV_, located just upstream of MCS 1 and MCS 2, and transiently introduced into HeLa cells. The transfected sensor cells initially showed a red fluorescent signal localized in the nucleus (Figure 2A, row 1). Sensor cells selectively responded to the cortisol treatment by translocating the red fluorescence into the cytoplasm (Figure 2A, row 2), whereas, cyproterone acetate (CPA), a structural analogue of cortisol, did not activate fluorescence translocation (Figure 2A, row 3). CPA is a passive antagonist of GR, which binds to GR but does not trigger nuclear translocation. The cortisol-mediated fluorescence translocation was inhibited when co-treated with CPA, indicating that the observed sensing event was induced by the specific binding of cortisol to GR (Figure 2A, row 4). The red-to-blue fluorescence intensity ratio (R/B FIR) was quantitatively analyzed before and after cortisol stimulation and showed the fluorescence cargos translocated to the cytoplasm in the presence of the target, cortisol (Figure 2A). We also prepared non-functioning mock sensor cells by transforming with the construct **m1**, containing a mutant I_N_, instead of construct **1**. The mock sensor cells did not respond to cortisol stimulation (Figure 2A, row 5), indicating that the observed fluorescence translocation in sensor cells can be attributed to split intein-mediated PTS. The distribution of red fluorescent cargo between the nucleus and the cytoplasm was analyzed by using the red fluorescence intensity ratio from the cytoplasm to the nucleus (R-FIR Cyto/Nuc) and R-FIR Cyto/Nuc of sensor cells and showed a three-fold increase when challenged with cortisol (Figure 2B). The formation of the PTS product was also verified with a Western blot analysis (Figure 2C). These results collectively showed that the sensor cells could detect and report the presence of cortisol through fluorescence translocation via CPS-mediated reconstitution and instantaneous activation of the signal peptide.

The performance of the sensor cells was, then, evaluated by measuring the limit of detection (LOD) and the response time of the developed sensor cells. To estimate the response rate, the developed sensor cells were treated with cortisol, and the increase in fluorescence translocation was monitored over time using fluorescence microscopy (Figure 2D and Figure S3). Signal translocation was observed as early as 1 min after cortisol stimulation, and R-FIR Cyto/Nuc reached a plateau after about 10 min. This represents remarkable improvements over the previously reported CPC-based reporting systems, which would enable practical screening of multiple analytes in a short time. The LOD was determined by monitoring the response of sensor cells when treated with cortisol in concentrations from 10 pM to 10 µM (Figure 2E). The increase in R-FIR Cyto/Nuc was observed with increasing cortisol concentrations of up to 10 nM before saturation. The sensor cells showed excellent sensitivity with an LOD of 3.5 nM, which was comparable to the recently reported LOD for de novo in vitro biosensors [21,34]. These results together demonstrate the excellent performance of sensitive and fast-responding sensor cells.

### 2.3. Differentiation of Cortisol Analogues Using Sensor Cells

**Figure 3:**
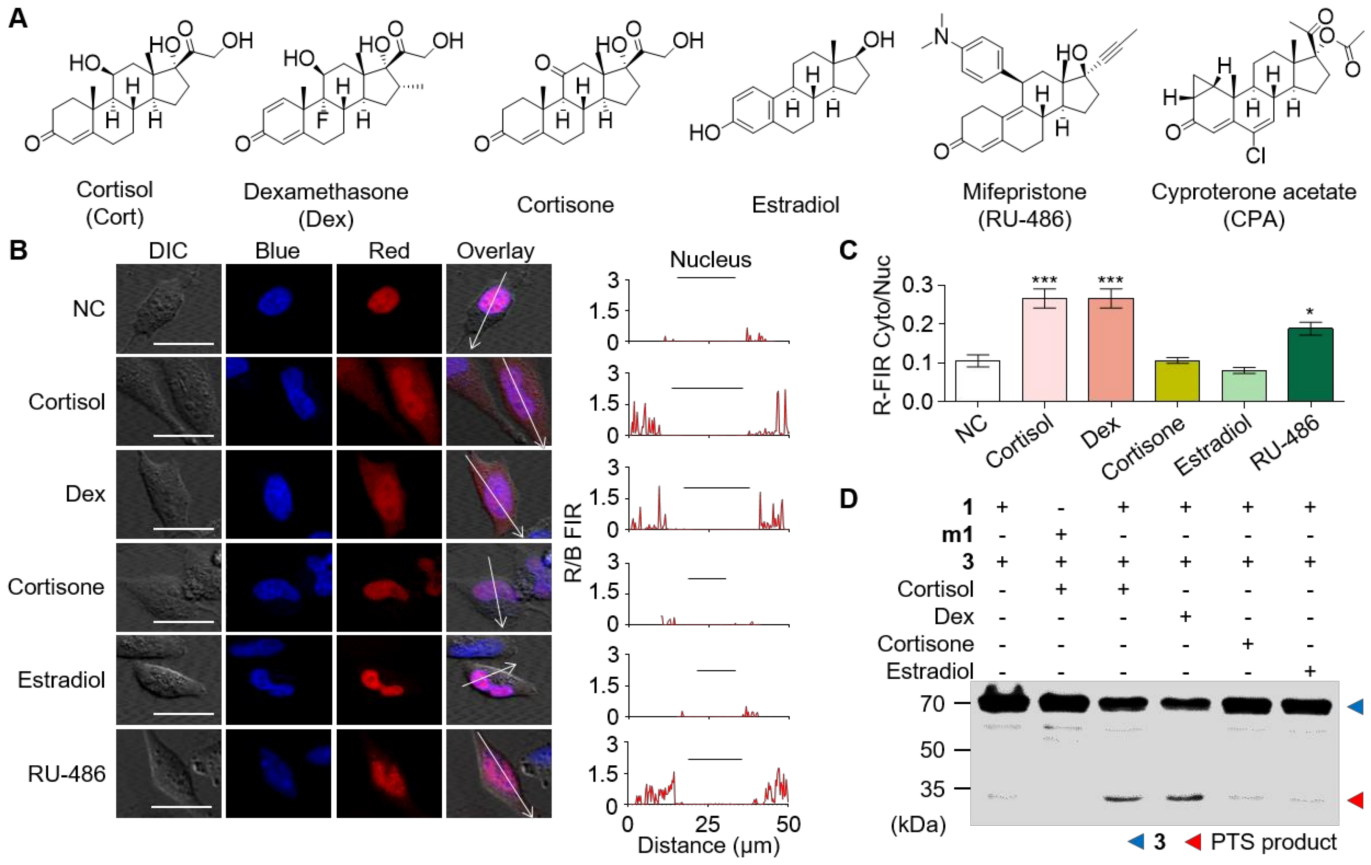
Discrimination of GR effectors from structural analogues of cortisol using the sensor cells. (**A**) The structures of cortisol and structural analogues are presented. Dexamethasone (Dex), mifepristone (RU-486), and cyproterone acetate (CPA) are an agonist, an active antagonist, and a passive antagonist of GR, respectively. Cortisone and estradiol are structural analogues of cortisol without GR binding activity; (**B**) the sensor cells responded to cortisol and the agonist and active antagonist of GR and discriminated the structural analogues (scale bar = 25 μm); (**C**) the R-FIR Cyto/Nuc are calculated (* *p* < 0.05 and *** *p* < 0.001). (**D**) The western blot analysis shows the formation of covalently conjugated active signal peptide when sensor cells are challenged with cortisol or its functional analogues.

Sensor cells were used to distinguish between the functional and structural cortisol analogues. Four different cortisol analogues, i.e., Dex, cortisone, estradiol, and RU-486, were selected (Figure 3A). Dex is a well-known cortisol agonist that binds to GR and prompts nuclear translocation, cortisone is an inactive form of cortisol, and estradiol is the major female sex hormone. While both cortisone and estradiol share high structural similarity with cortisol, neither steroid hormone binds to GR [35,36]. RU-486 is an active GR antagonist that induces GR translocation to the nucleus but inhibits transcriptional activation [37,38,39]. The sensor cells were individually treated with Dex, cortisone, estradiol, and RU-486 and monitored using fluorescence microscopy (Figure 3B). The sensor cells treated with Dex or RU-486 showed fluorescence translocation comparable to cortisol, whereas cortisone- or estradiol-treated sensor cells showed negligible fluorescence in the cytoplasm (Figure 3B,C). This result shows that the sensor cells can identify GR effectors and monitor GR dynamics under complex cellular regulations. Thus, these sensor cells could provide a plausible tool for screening of GR effectors that can improve the therapeutic index related to hyper- or hypocortisolism.

### 2.4. Screening of Natural Products for GR Effectors Using Sensor Cells

**Figure 4:**
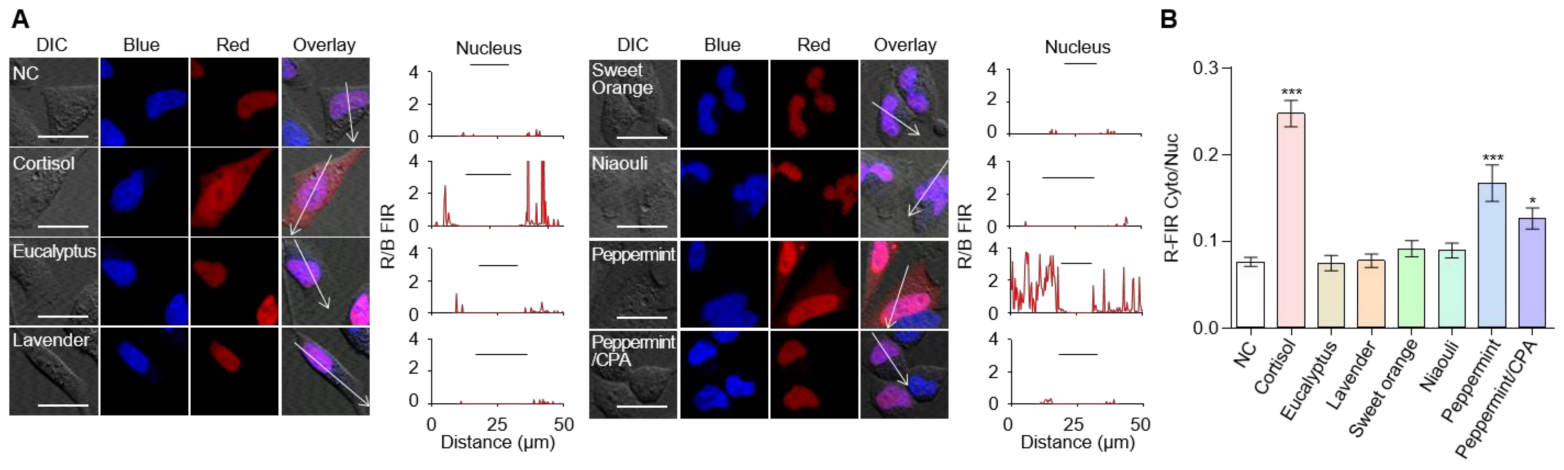
Screening for GR effectors in essential oil extracts. (**A**) The sensor cells were treated with 5 different essential oil extracts. Sensor cells treated with peppermint oil showed red fluorescence translocation which was repressed by co-treatment with CPA (scale bar = 25 μm); (**B**) the R-FIR Cyto/Nuc are calculated (* *p* < 0.05 and *** *p* < 0.001).

Given that the developed sensor cells could screen unknown analytes in complex media, based on their biological functions, we screened for natural mimetics of cortisol in essential oils. Essential oils were selected because they have often been used in stress relieving therapies [40]. We chose five common essential oils extracted from eucalyptus, lavender, sweet orange, niaouli, or peppermint. The sensor cells were treated with each of the five essential oils and individually monitored using fluorescence microscopy (Figure 4). The fluorescence intensity profiles of the cytoplasm and the nucleus were analyzed (Figure 4A,B). Among the five oils tested, peppermint oil showed a strong response. The peppermint oil-induced signal was significantly suppressed when co-treated with CPA, implying that the observed signal originated from the specific binding of GC-like molecules, GR effectors, present in the peppermint oil.

**Figure 5:**
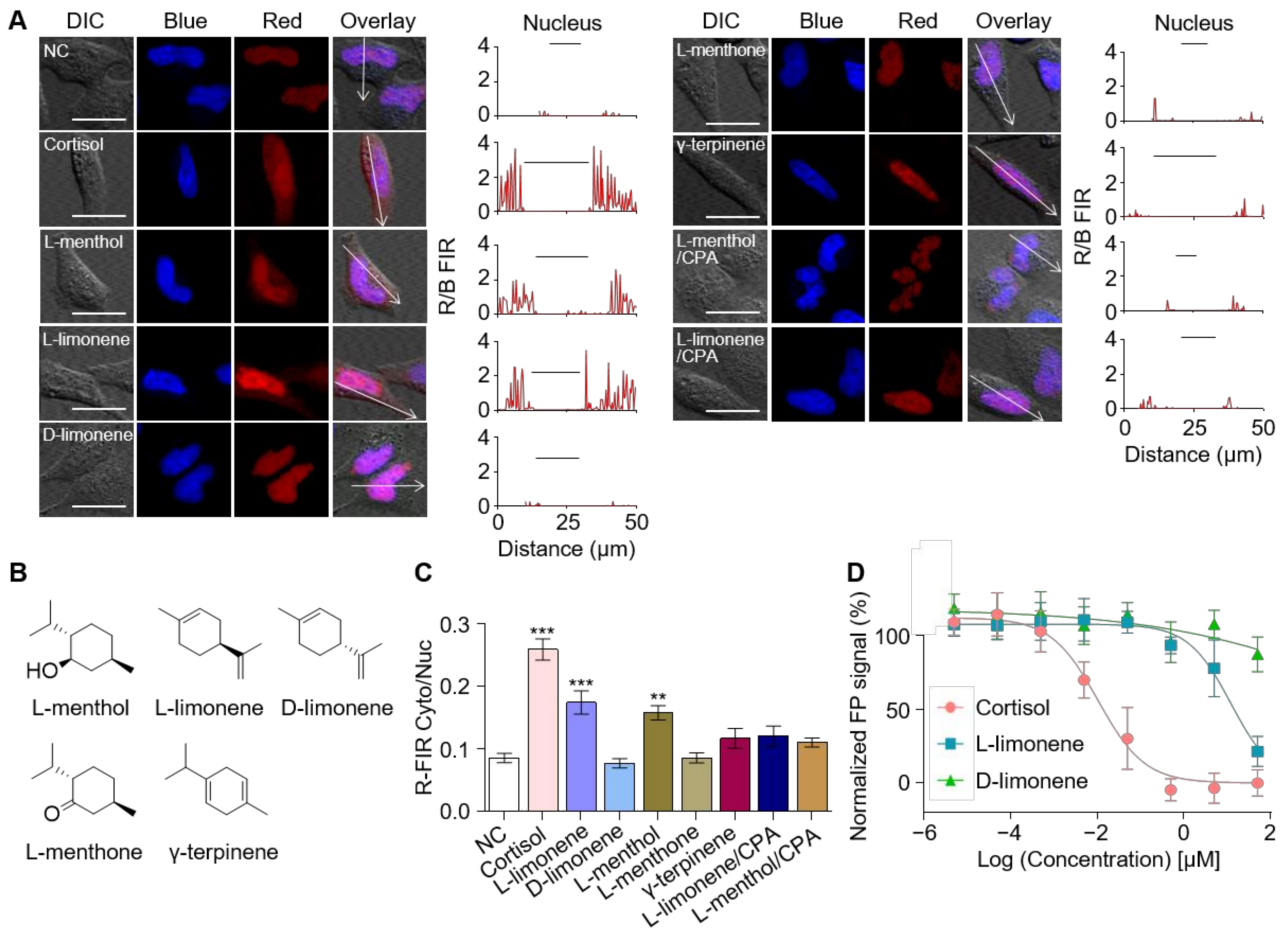
Identification of GR effectors from the components of peppermint oil extracts using sensor cells. (**A**) The sensor cells responded to L-limonene and L-menthol, whose signals are repressed on co-treatment with CPA (scale bar = 25 μm); (**B**) the structures of the major components of peppermint oil, i.e., L-limonene, L-menthol, L-menthone, and γ-terpinene are prominent components of peppermint oil. D-limonene is an enantiomer of L-limonene; (**C**) the R-FIR Cyto/Nuc ratios are calculated (** *p* < 0.01 and *** *p* < 0.001); (**D**) In vitro interaction between GR and L-limonene was studied using a competitive GR binding assay based on fluorescence polarization.

To identify GR effectors, the sensor cells were challenged with four major components of peppermint oil, namely, L-menthol, L-limonene, L-menthone, and γ-terpinene, individually (Figure 5A–C). These four components were selected by considering the abundance as well as their known functions (Table S1) [41,42,43]. The sensor cells responded to L-limonene and L-menthol treatment; the signals induced by these were significantly suppressed on co-treatment with CPA (Figure 5A,C), indicating that these substances selectively bind to and activate GR. The anti-inflammatory activities of limonene and L-menthol have been previously reported; however, to the best of our knowledge, their association with GR-mediated gene regulation has not been determined [44,45]. The stereoisomer of L-limonene (for example, d-limonene), did not induce fluorescence translocation, indicating that the sensor cells could distinguish the non-functional stereoisomer.

Of interest, both the pairs, L-menthol/L-menthone and cortisol/cortisone, are structurally homologous. Menthol has a structure similar to the C ring of cortisol, whereas menthone has a structure similar to the C ring of cortisone (Figure 3A and Figure 5B). The common difference they share is that the hydroxyl group in menthol and cortisol is substituted by a carbonyl group in menthone and cortisone. As GR forms a hydrogen bond with C_11_-OH in cortisol for ligand binding, this change may result in reduced binding activity via elimination of the hydrogen bonding [46,47].

### 2.5. Further Analysis for Determining the GR Effector Functions

We further analyzed the newly identified effector, L-limonene, to better understand its function as a GR effector. Cell-based assays ruled out the passive antagonistic role of L-limonene given L-limonene transported GR to the nucleus. The in vitro competitive GR binding assay using fluorescence polarization also supported this result by demonstrating that L-limonene bound to GR (Figure 5C).

**Figure 6:**
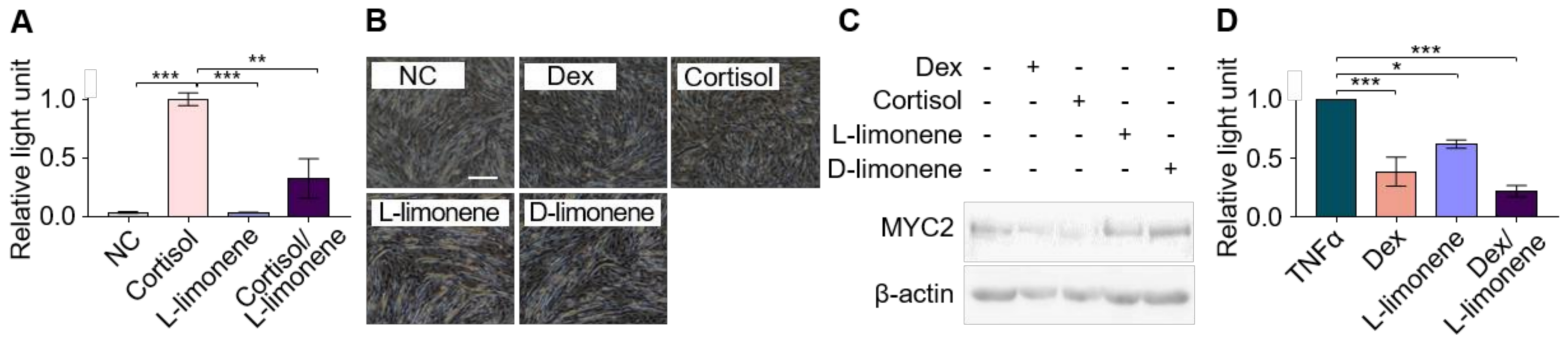
Elucidating the function of L-limonene as a GR-effector. (**A**) GR-mediated transactivation was investigated using a glucocorticoid response element (GRE)-based reporter gene assay, and L-limonene did not show GR-agonist-like activity; (**B**) the muscle atrophy effect was analyzed by monitoring morphologic differences of differentiated C2C12 cells, Dex and Cort inhibited the differentiation of C2C12 cells, whereas L-limonene treated cells differentiated successfully (scale bar = 200 μm); (**C**) the expression level of the myosin heavy chain II (MYC2) was measured using Western blotting, the expression of MYC2 was inhibited in Dex and Cort-treated cells, whereas L-limonene did not suppress MYC2 expression; (**D**) the reporter gene assay was performed to monitor the GR-mediated tethered indirect transrepression effect of L-Limonene (* *p* < 0.05, ** *p* < 0.01 and *** *p* < 0.001).

While the sensor cells are able to screen for GR effectors which bind to GR and prompt nuclear translocation, these GR effectors include agonists, active antagonists, and SEGRA. As each effector induces different GR-mediated responses, further analysis is required to reveal the function of these effectors. At first, a reporter gene assay was carried out to test GR-mediated transactivation via luminescence [48]. Although this assay requires the use of luciferin, a small molecule whose effect might need to be considered in data interpretation, it provides valuable information to validate our result. L-limonene did not induce GR-mediated transactivation; in contrast, it significantly inhibited transactivation induced by cortisol treatment (Figure 6A). This result precludes the GR agonistic function of L-limonene, indicating that it could be an active antagonist or a SEGRA. Using the mouse myoblast cell line C2C12, we determined the muscle atrophy effect of L-limonene. The results showed that L-limonene does not cause the well-recognized adverse effect of GR agonist-mediated transactivation (Figure 6B). A Western blot analysis confirmed regular expression of the myosin heavy chain II (MYC2) on treatment of L-limonene, whereas treatment with cortisol or Dex significantly inhibited MYC2 expression (Figure 6C and Figure S5); this also indicates that L-limonene is not a GR agonist.

We finally studied the effect of L-limonene in a GR-mediated tethered indirect transrepression, which is a known function of a SEGRA. The result showed that L-limonene repressed the TNF-α mediated expression of inflammatory genes, indicating that it functions as a SEGRA (Figure 6D). Limonene has been previously suggested as an inhibitor of nitric oxide and prostaglandin E2 production with a potent anti-inflammatory activity [45,49]. Our results indicate that L-limonene likely controls the selective induction of NO synthase and cyclooxygenase-2 genes via downregulation of NF-κB signaling.

## 3. Materials and Methods

### 3.1. General Procedures

General chemicals of the best grade available were supplied by Sigma-Aldrich (St. Louis, MO, USA) and Fisher Scientific (Pittsburgh, PA, USA). DNA primers were purchased from MBiotech (Hanam, Korea). Restriction enzymes were purchased from Elpis Biotech (Daejeon, Korea) and New England Biolabs (Ipswich, MA, UK). Lavender oil was purchased from Sigma-Aldrich (St. Louis, MO, USA); peppermint oil, eucalyptus oil, and sweet orange oil were purchased from Bleu Lavande (Quebec, Canada), and Niaouli oil was purchased from Pranarom (Ghislenghien, Belgium). Dulbecco’s modified Eagle’s medium (DMEM), penicillin-streptomycin, trypsin-EDTA, and fetal bovine serum (FBS) were purchased from Welgene (Daegu, Korea). Cortisol, Dex, cortisone, estradiol, RU-486, L-limonene, D-limonene, and γ-terpinene were purchased from Sigma-Aldrich (St. Louis, MO, USA). Cyproterone acetate (CPA) was purchased from TCI Chemicals (Tokyo, Japan). L-menthone was purchased from HWI Group (Rülzheim, Germany), and L-menthol was purchased from Alfa Aesar (Haverhill, MA, USA). Confocal fluorescence images were obtained using an Eclipse Ti confocal microscope from Nikon Instruments (Tokyo, Japan) with excitation wavelengths of 358, 488, and 594 nm and corresponding emission filters. The fluorescence intensity was analyzed using the Nikon NIS-Element BR 4.60 software from Nikon Instruments (Tokyo, Japan) and ImageJ software from U.S. National Institutes of Health (Bethesda, MD, USA).

### 3.2. Construct Design and DNA Cloning

DNA cloning was performed according to the standard protocols. All constructed plasmids were confirmed using DNA sequencing and amplified using the E. coli strain DH5α. The mCherry gene with 3’-end SacII enzyme site was introduced into a pIRES vector between the NotI and SalI sites. Then, the mNES_N_-Npu_N_-NLS gene was inserted between the SacII and Xba I sites to create a gene encoding Cherry-mNES_N_-Npu_N_-NLS, construct **1**. The gene encoding GR was inserted into pBI-CMV1 between the NheI and XhoI sites. Then, Npu_C_-mNES_C_ was inserted between XhoI and MluI to create construct **2** (GR-Npu_C_-mNES_C_). The construct **1** was inserted into the pBI-CMV1 containing construct **2** between the EcoRI and XbaI sites to create a pRJH013 plasmid. In addition, the GR-Npu_C_-mNES_C_ gene was inserted into the pET28a vector between the NdeI and NheI sites and a synthetic 2xFLAG gene was inserted between NheI and NotI sites to create construct **3** (GR-Npu_C_-mNES_C_-2xFLAG). Then, the GR-Npu_C_-mNES_C_-2xFLAG gene was introduced into the pBI-CMV1 vector containing construct **1** between the BamHI and MluI sites to create a pRJH022 plasmid. Then, C1A point mutation of Npu_N_ was introduced into pRJH013 and pRJH022 to generate **m1** containing pRJH025 and pRJH026, respectively, using the QuickChange Site-Directed Mutagenesis kit.

### 3.3. Screening of Cortisol and GR Effectors Using Sensor Cells

HeLa cells were grown in 35 mm confocal dishes and transiently transfected using a pRJH013 plasmid. Protein expression was allowed to proceed at 37 °C for 24 h, and the sensor cells were treated with analytes. The LOD is determined as the concentration at which signal ratio is thrice the standard deviation of the mean of blank determinations. The cells were washed with PBS, and their nuclei were stained with Syto9 (3 µM) or Hoechst 33342 (8 µM). Then, the fluorescence images were obtained to monitor the fluorescence signal translocation. At least 3 sets of experiments were performed, and approximately 30 cells were analyzed in each experiment. Fluorescence intensity of each cell compartment was analyzed using ImageJ software.

### 3.4. Fluorescence Polarization-Based GR Competitor Assay

GR binding affinity was measured with PolarScreen GR competitor assay Red from Thermo Fisher Scientific (Waltham, MA, USA), according to the manufacturer’s instructions. Fluorescence polarization was measured using a Tecan Spark microplate reader (Männedorf, Switzerland).

### 3.5. Studying Muscle Atrophy in the Presence of GR Effectors and Western Blot Analysis

C2C12 myoblasts were grown in 35 mm dishes (1 × 10^5^) before differentiation. Differentiation was induced when myoblasts reached 80% confluence by decreasing FBS to 2% in the presence of Dex, cortisol, L-limonene, or D-limonene (100 μM), individually. The morphologic differences between the different groups were photographed with a Nikon Eclipse Ts2 microscope (Tokyo, Japan) over 4 days. The experiment was repeated three times. Western blotting was performed to determine the expression level of the myosin heavy chain II (MYC2) using an anti-MYC2 antibody and anti-β-actin antibody from Santa Cruz Biotechnology (Dallas, TX, USA). The Western blots were developed with the ECL system from Thermo fisher Scientific (Waltham, MA, USA), according to the manufacturer’s protocols and imaged using ImageQuant LAS 500 from GE Healthcare (Chicago, IL, USA).

### 3.6. Reporter Gene Assay

HeLa cells were grown in a 96-well white plate (1 × 10^4^ cells/well) in DMEM containing 5% FBS without phenol red for 24 h and transfected with the pGL4.36 [luc2P/MMTV/hygro] vector or the pGL4.32 [luc2P/NF-κB-RE/hygro] vector from Promega (Madison, WI, USA). Cells were treated with analytes, as indicated in the figure legends, after which luciferase assays were performed according to manufacturer’s protocol.

### 3.7. Statistics

All data are presented as means and standard errors of mean. Data were analyzed, and graphs were plotted using GraphPad Prism 5.0 (San Diego, CA, USA).

## 4. Conclusions

In this study, we developed a new fast-responding cortisol sensor cell for the screening of GR effectors. The developed sensor cells utilized the reconstitution of signal peptide, which is instantly activated via CPS, and consequent translocation of AFP. Signal peptides do not require folding into a functional tertiary structure to regain their activity, and they cannot be activated via simple binding of the two split fragments, which minimizes false-positive or false-negative signals. Moreover, this approach does not require an external source of energy or cofactors which could interfere with the targets’ function and compromise sensor fidelity.

The developed sensor cells could report the presence of cortisol and discriminate between its analogues with excellent sensitivity within a short response time. The sensor cells could successfully screen potential GR effectors from essential oil and identified novel GR-effectors, L-limonene and L-menthol, present in peppermint oil. We further studied the properties of L-limonene and suggested that L-limonene is possibly a SEGRA. SEGRAs could function as anti-inflammatory agents via transrepression of NF-κB signaling without causing the adverse effects known to be associated with GR agonists such as muscle wasting with long-term or high-dosage use. The developed cortisol sensor cell provided a novel way to screen GR effectors under complex cellular regulation conditions, which promises better efficacy of the identified drug candidates as compared with in vitro assays.

## Data Availability

The data presented in this study are included in this published article and its additional files. All the data can be shared upon request by email.

## Supplementary Materials

The following are available online at https://www.mdpi.com/article/10.3390/ijms22094747/s1, Figure S1: Tests of designed recombinant NES for the sensor cells based on the NES database, Figure S2: Translocation caused by solvents used for dissolving different materials, Figure S3: Time-dependent translocation after cortisol treatment of sensor cells, Figure S4: Western blot quantification of relative protein level by ImageJ, Figure S5: A time-response curve of Cort- or L-limonene stimulation of sensor cells, Table S1: Components of peppermint essential oil.
