# Supplementary material for: Rapid Screening of Glucocorticoid Receptor (GR) Effectors Using Cortisol-Detecting Sensor Cells
Source: Int J Mol Sci. 2021 Apr 29;22(9):4747. doi: 10.3390/ijms22094747 (PMC8124850; doi:10.3390/ijms22094747)
Supplement: Supplementary file 1 [file ijms-22-04747-s001.zip › ijms-1189763-supplementary.pdf]

Supporting Information

Rapid screening of glucocorticoid receptor (GR) effectors using cortisol-detecting sensor cells

Jeahee Ryu†, Euiyeon Lee†, Chungwon Kang†, Minhyeong Lee, Soyoun Kim, and Youngeun Kwon*

Department of Biomedical Engineering, Dongguk University, Seoul, Korea

*Corresponding author: ykwon@dongguk.edu

†These authors contributed equally.

Table of contents **Page #**

Supplementary methods

Plasmid Construction 2
Protein sequence 3

Supplementary Figures

Figure S1 4
Figure S2 4
Figure S3 5
Figure S4 5
Figure S5 6

Supplementary Table 1 6

Plasmid construction

DNA cloning was performed according to standard protocols. The sequences of all plasmids were confirmed by DNA sequencing and amplified using the *E. coli* strain DH5α.

mCherry (pJHJ066): The PCR-amplified mCherry gene was inserted into pBI-CMV1 between the EcoRI and ApaI sites to create the pJHJ066 plasmid.

mNES-mCherry (pRJH006): The synthetic DNA sequence encoding for mNES (KVYPIILRL*CFN*LSL) derived from the human SARS coronavirus ORF-9b protein was inserted into the pJHJ066 vector containing the mCherry gene between the *Apa*I and *Xba*I sites.

mCherry-mNES$_N$-Npu$_N$-NLS (pRJH014): The mCherry gene was amplified from pJHJ066 and was inserted into the pIRES vector between the NotI and SalI sites to create the pRJH007 vector. The synthetic mNES$_N$-Npu$_N$-NLS gene was inserted into pRJH007 between the SacII and XbaI sites to create pRJH009. Finally, the mCherry-mNES$_N$-Npu$_N$-NLS sequence was amplified from pRJH009 and was inserted into pBI-CMV1, between the EcoRI and XbaI sites to create pRJH014.

mCherry-mNES$_N$-Npu$_N$-NLS and GR-Npu$_C$-mNES$_C$ (pRJH013): The gene encoding for GR was PCR amplified using pKCW001, including the GR gene sequence gathered from Human Pituitary Gland QUICK-Clone cDNA, as a template [1], and then was inserted into the pRJH014 vector between the NheI and XhoI sites to create pRJH008. The synthetic Npu$_C$-mNES$_C$ gene was inserted into the XhoI and MluI site of pRJH008 to create pRJH010. Finally, GR-Npu$_C$-mNES$_C$ encoding cDNA was amplified from pRJH010 and was inserted into the pRJH014 between the NheI and EagI sites to create pRJH013.

mCherry-mNES$_N$-Npu$_N$-NLS and GR-Npu$_C$-mNES$_C$-2xFLAG (pRJH022): The PCR amplified GR-Npu$_C$-mNES$_C$ encoding sequence (template: pRJH013) was inserted into the pET28a vector between the NdeI and NheI sites to create pRJH019. The synthetic DNA oligomer encoding the flag-flag sequence was inserted between the NheI and NotI sites of pRJH019 to create pRJH020. Finally, GR-Npu$_C$-NES$_C$-2xFLAG gene was amplified using primer set 6 (template: pRJH020) and inserted into pRJH014 using the BamHI and MluI sites to create pRJH022.

mCherry-NES$_N$-mNpu$_N$-NLS and GR-Npu$_C$-NES$_C$ (pRJH025): The C1A point mutation of Npu$_N$ was introduced into pRJH013 using PCR, with the QuikChange Site-Directed Mutagenesis kit (Agilent, Santa Clara, CA) to generate pRJH025. The mutagenetic oligonucleotides are 5'-

CGATTATTCTGCGTCTGGCCTTGAGCTACGAAACGG-3’ and 5’-CCGTTTCGTAGCTCAAGGCCAGACGCAGAATAATCG-3’.

mCherry-NES$_N$-mNpu$_N$-NLS and GR-Npu$_C$-NES$_C$-2xFLAG (pRJH026): The C1A point mutation of Npu$_N$ was introduced into pRJH022 using PCR, using the QuikChange Site-Directed Mutagenesis kit (Agilent, Santa Clara, CA) to generate pRJH026. The mutagenetic oligonucleotides are same as above.

Protein sequence

GR-Npu$_C$-mNES$_C$ (1)
Expected Mass (kDa): 90.57
Sequence:
MDSKESLTPGREENPSSVLAQERGDVMDFYKTLRGGATVKVSASSPSLAVASQSDSKQRRLLVDFPKGSVSNAQQP
DLSKAVSLSMGLYMGETETKVMGNDLGFPQQGQISLSSGETDLKLLEESIANLNRSTSVPENPKSSASTAVSAAPTEK
EFPKTHSDVSSEQQHLKGQTGTNGGNVKLYTTDQSTFDILQDLEFSSGSPGKETNESPWRSDLLIDENCLLSPLAGE
DDSFLLEGNSNEDCKPLILPDTKPKIKDNGDLVLSSPSNVTLPQVKTEKEDFIELCTPGVIKQEKLGTVYCQASFPGANI
IGNKMSAISVHGVSTSGGQMYHYDMNTASLSQQQDQKPIFNVIPPIPVGSENWNRCQGSGDDNLTSLGTLNFPG
RTVFSNGYSSPSMRPDVSSPPSSSSTATTGPPPKLCLVCSDEASGCHYGVLTCGSCKVFFKRAVEGQHNYLCAGRND
CIIDKIRRKNCPACRYRKCLQAGMNLEARKTKKKIKGIQQATTGVSQETSENPGNKTIVPATLPQLTPTLVSLLEVIEPE
VLYAGYDSSVPDSTWRIMTTLNMLGGRQVIAAVKWAKAIPGFRNLHLDDQMTLLQYSWMFLMAFALGWRSYRQ
SSANLLCFAPDLIINEQRMTLPCMYDQCKHMLYVSSELHRLQVSYEEYLCMKTLLLLSSVPKDGLKSQELFDEIRMTYI
KELGKAIVKREGNSSQNWQRFYQLTKLLDSMHEVVENLLNYCFQTFLDKTMSIEFPEMLAEIITNQIPKYSNGNIKKL
LFHQKLEIKIATRKYLGKQNVYDIGVERDHNFALKNGFIASNCFNLSL

mCherry-mNES$_N$-Npu$_N$-NLS (2)
Expected Mass (kDa): 44.29
Sequence:
MVSKGEEDNMAIIKEFMRFKVHMEGSVNGHEFEIEGEGEGRPYEGTQTAKLKVTKGGPLPFAWDILSPQFMYGSK
AYVKHPADIPDYLKLSFPEGFKWERVMNFEDGGVVTVTQDSSLQDGEFIYKVKLRGTNFPSDGPVMQKKTMGWE
ASSERMYPEDGALKGEIKQRLKLKDGGHYDAEVKTTYKAKKPVQLPGAYNVNIKLDITSHNEDYTIVEQHERAEGRH
STGGMDELYKPRKVYPIILRLCLSYETEILTVEYGLLPIGKIVEKRIECTVYSVDNNGNIYTQPVAQWHDRGEQEVFEYC
LEDGSLIRATKDHKFMTVDGQMLPIDEIFERELDLMRVDNLPNIKIATRKYLGKQNVYDIGVERKRPAATKKAGQAK
KKKLD

GR-Npu$_C$-mNES$_C$-2xFLAG (3)
Expected Mass (kDa): 92.72
Sequence:
MDSKESLTPGREENPSSVLAQERGDVMDFYKTLRGGATVKVSASSPSLAVASQSDSKQRRLLVDFPKGSVSNAQQP
DLSKAVSLSMGLYMGETETKVMGNDLGFPQQGQISLSSGETDLKLLEESIANLNRSTSVPENPKSSASTAVSAAPTEK
EFPKTHSDVSSEQQHLKGQTGTNGGNVKLYTTDQSTFDILQDLEFSSGSPGKETNESPWRSDLLIDENCLLSPLAGE
DDSFLLEGNSNEDCKPLILPDTKPKIKDNGDLVLSSPSNVTLPQVKTEKEDFIELCTPGVIKQEKLGTVYCQASFPGANI
IGNKMSAISVHGVSTSGGQMYHYDMNTASLSQQQDQKPIFNVIPPIPVGSENWNRCQGSGDDNLTSLGTLNFPG
RTVFSNGYSSPSMRPDVSSPPSSSSTATTGPPPKLCLVCSDEASGCHYGVLTCGSCKVFFKRAVEGQHNYLCAGRND

CIIDKIRRKNCPACRYRKCLQAGMNLEARKTKKKIKGIQQATTGVSQETSENPGNKTIVPATLPQLTPTLVSLLEVIEPE
VLYAGYDSSVPDSTWRIMTTLNMLGGRQVIAAVKWAKAIPGFRNLHLDDQMTLLQYSWMFLMAFALGWRSYRQ
SSANLLCFAPDLIINEQRMTLPCMYDQCKHMLYVSSELHRLQVSYEEYLCMKTLLLLSSVPKDGLKSQELFDEIRMTYI
KELGKAIVKREGNSSQNWQRFYQLTKLLDSMHEVVENLLNYCFQTFLDKTMSIEFPEMLAEIITNQIPKYSNGNIKKL
LFHQKLEIKIATRKYLGKQNVYDIGVERDHNFALKNGFIASNCFNLSLASDYKDDDDKDYKDDDDK

mCherry-mNES$_N$-mNpu$_N$-NLS (m1)
Expected Mass (kDa): 44.25
Sequence:
MVSKGEEDNMAIIKEFMRFKVHMEGSVNGHEFEIEGEGEGRPYEGTQTAKLKVTKGGPLPFAWDILSPQFMYGSK
AYVKHPADIPDYLKLSFPEGFKWERVMNFEDGGVVTVTQDSSLQDGEFIYKVKLRGTNFPSDGPVMQKKTMGWE
ASSERMYPEDGALKGEIKQRLKLKDGGHYDAEVKTTYKAKKPVQLPGAYNVNIKLDITSHNEDYTIVEQHERAEGRH
STGGMDELYKPRKVYPIILRLALSYETEILTVEYGLLPIGKIVEKRIECTVYSVDNNGNIYTQPVAQWHDRGEQEVFEYC
LEDGSLIRATKDHKFMTVDGQMLPIDEIFERELDLMRVDNLPNIKIATRKYLGKQNVYDIGVERKRPAATKKAGQAK
KKKLD

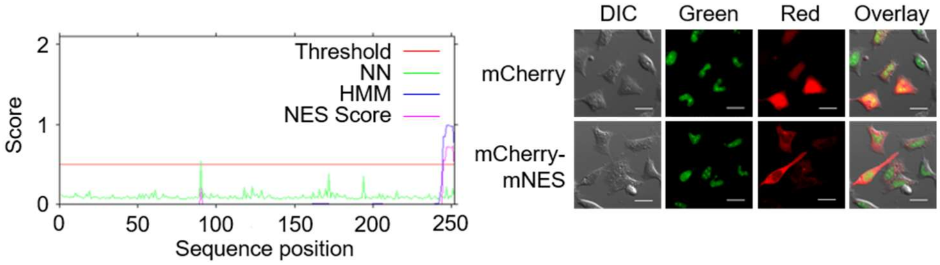

Figure S1. Tests of designed recombinant NES for the sensor cells based on the NES database. The delivery of an autofluorescent protein into the cytoplasm by modified NES (mNES) was confirmed by monitoring the cellular localization of the mCherry-mNES fusion protein (scale bar = 25 um).

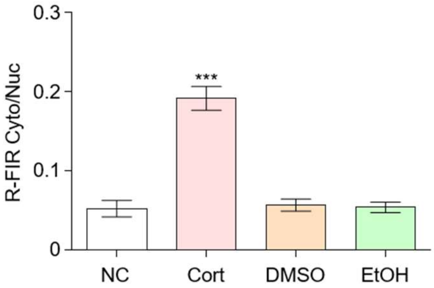

Figure S2. Translocation caused by solvents used for dissolving different materials. DMSO and EtOH showed no significant increase in translocation among treatments. Data were analyzed using 1-way ANOVA with the Tukey multiple comparison test (***p<0.001).

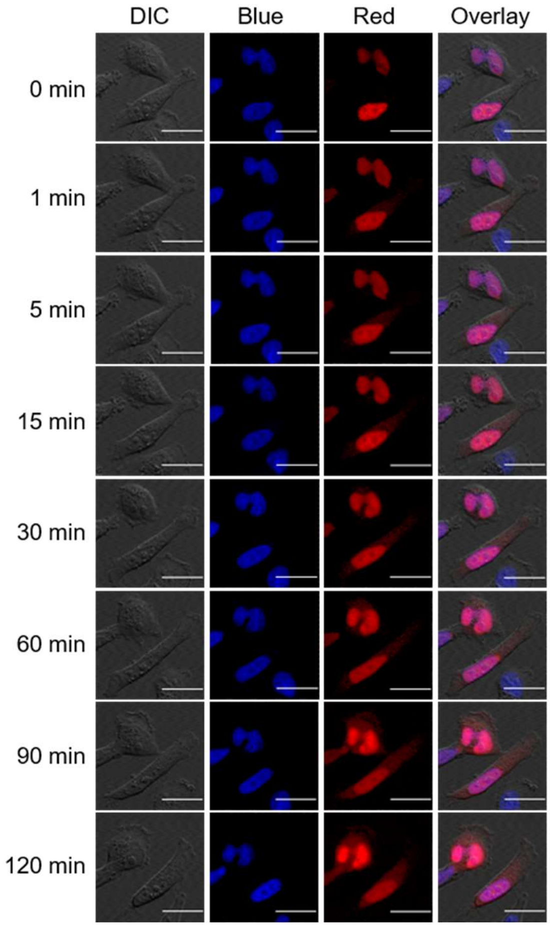

Figure S3. Time-dependent translocation after cortisol treatment of sensor cells. Fluorescent translocation started within 1 minute after stimulation (scale bar = 25 um).

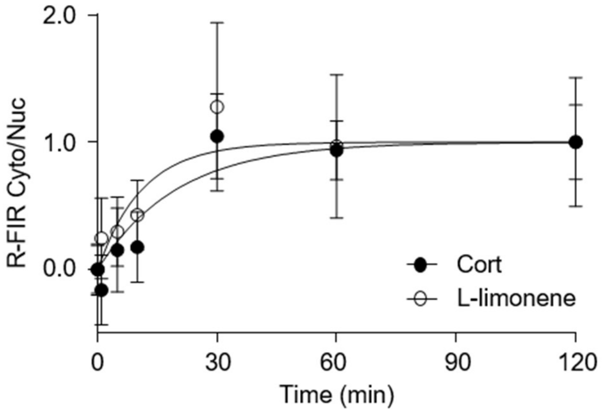

Figure S4. A time-response curve of Cort- or L-limonene stimulation of sensor cells. The sensor cells were treated with 10 μM each of Cort and L-limonene for time-dependent measurements. The reaction rates of Cort and L-limonene did not differ with time.

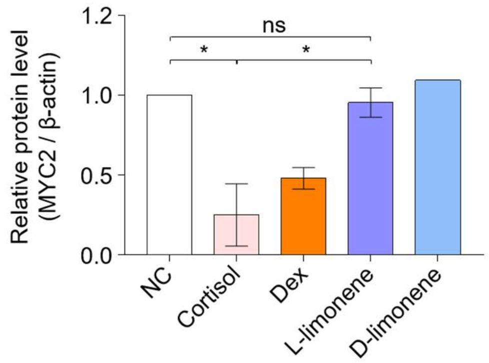

Figure S5. Western blot quantification of relative protein level by ImageJ. Expression of MYC2 was normalized to β-actin expression.

Table S1. Components of peppermint essential oil *[2]*

Components	%	Components	%
Menthol	38.45	Sabinene	0.43
Menthone	21.8	Linalool	0.37
1,8-Cineole	5.62	α-Terpinene	0.35
Neo-Menthol	4.19	Cis-β-Ocimene	0.34
Isomenthone	3.75	Para-Cymene	0.32
Menthofuran	2.08	3-Octanol	0.26
Limonene	1.58	β-Myrcene	0.20
Trans-Sabinene Hydrate	0.86	α-Terpinolene	0.18
β-Pinene	0.81	3-Octanol Acetate	0.15
α-Pinene	0.56	Cis-Sabinene Hydrate	0.11
Gamma-Terpinene	0.56	3-Octanol Acetate	0.05

References

1. Jeon, H.; Lee, E.; Kim, D.; Lee, M.; Ryu, J.; Kang, C.; Kim, S.; Kwon, Y. Cell-based biosensors based on intein-mediated protein engineering for detection of biologically active signaling molecules. *Anal. Chem.* 2018, *90*(16), 9779-9786.
2. Wu, Z.; Tan, B.; Liu, Y.; Dunn, J.; Martorell Guerola, P.; Tortajada, M.; Cao, Z.; Ji, P. Chemical composition and antioxidant properties of essential oils from peppermint, native spearmint and scotch spearmint. *Molecules* 2019, *24*, 2825.
